# P-1937. Does Microbiological Clearance Impact Outcomes in Patients Contracting Candida auris?

**DOI:** 10.1093/ofid/ofaf695.2105

**Published:** 2026-01-11

**Authors:** Hafsa Khan Tareen, Samavia Mehmood, Joveria Farooqi, Sadaf Zaka, Kauser Jabeen

**Affiliations:** Aga Khan University, Multan, Punjab, Pakistan; Aga khan hospital, karachi, Sindh, Pakistan; Agakhan University Hospital, Karachi, Sindh, Pakistan; Aga Khan University Hospital, Karachi, Sindh, Pakistan; Agakhan University Hospital, Karachi, Sindh, Pakistan

## Abstract

**Background:**

The CDC reported an increase in *C. auris* cases from 44% to 95% between 2019-2021. An estimated 41% of *Candida auris* isolates are already resistant to >2 antifungals. Since its first report in 2014, recent data on the trend of *C. auris* fungemia from Pakistan is not readily available. This data assessed changes in epidemiology, antifungal resistance, and clinical outcomes of *C. auris* fungemia.

**Table:**
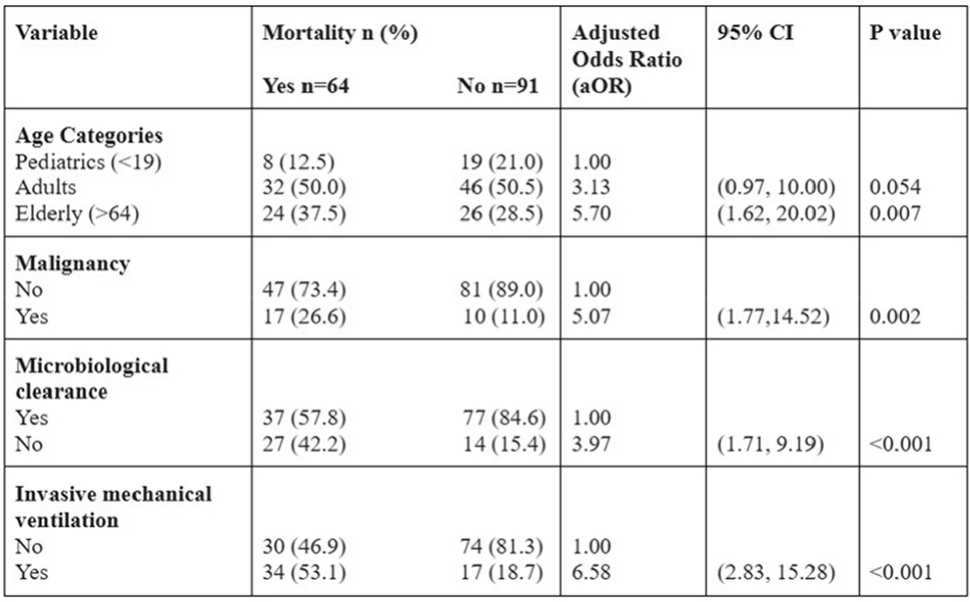
Multivariable analysis with adjusted Odds Ratios (aOR)

**Methods:**

Retrospective cross-sectional study was conducted at Aga Khan University Hospital, Karachi, from 2017 to 2024. Clinical outcomes included mortality, length of hospital and ICU stay, and microbiological failure (defined as persistent positive blood cultures beyond seven days of antifungal therapy). Data on clinical characteristics included comorbidities, immunosuppression, prior antimicrobial exposure, MDR co-infections, and surgical interventions.

**Results:**

155 patients were included, with a median age of 54.0 years (IQR: 26.0–70.0), 60.6% of which were males, median length of hospital stay was 13.0 days (IQR: 4.0–24.0), and ICU stay was 17.5 days (IQR: 5.0–30.0) with 32.9% requiring mechanical ventilation. Median antifungal duration was 7.0 days (IQR: 5.0–12.0). The MIC50 and MIC90 was 64.0 µg/mL, 256µg/mL for fluconazole; 1.0 µg/mL,1.0 µg/mL for amphotericin; and 0.12 µg/mL, 0.25 µg/mL for anidulafungin. Resistance to fluconazole was noted in 94.3% of the tested isolates, while all tested isolates remained sensitive to amphotericin and anidulafungin. Most patients stayed in the hospital for a duration of 1–2 weeks (38.7%, n=60). Hypertension (46.5%, n=72) and diabetes mellitus (40.0%, n=62) were the most frequently reported comorbidities. Over one-third (36.8%) had ≥2 positive blood cultures. Prior antibiotic and antifungal exposure were 94.2% and 29.0%, respectively. Crude mortality was 41.3% (n=64).

**Conclusion:**

While earlier studies have compared mortality between *C. auris* and non-*C. auris* candidemia as a newly emerging disease, we have tried to explore the factors associated with death in *C. auris* candidemia patients. Lack of microbiologic clearance had a fourfold increased risk of inpatient mortality (aOR: 3.97). Older age (aOR: 5.7), malignancy (aOR: 5.07), and invasive mechanical ventilation (aOR: 6.58) emerged as strong independent predictors of mortality.

**Disclosures:**

All Authors: No reported disclosures

